# GVC transformation and a new investment landscape in the 2020s: Driving forces, directions, and a forward-looking research and policy agenda

**DOI:** 10.1057/s42214-020-00088-0

**Published:** 2021-02-02

**Authors:** James X. Zhan

**Affiliations:** United Nations Conference on Trade and Development, Geneva, Switzerland

**Keywords:** investment, trade, technological change, global value chains, MNEs, research, policy, sustainable development

## Abstract

Global value chains (GVCs) will undergo substantive transformation in the decade ahead, reshaping the global trade and investment landscape. The change will be driven by five major forces: economic governance realignment, the new industrial revolution, the sustainability endeavor, corporate accountability, and resilience-oriented restructuring. All of this will present challenges and opportunities for firms and states alike, leading to an investment-development paradigm shift. This article discusses the five driving forces for the GVC transformation, projects ten broad trends in the evolution of the global trade and investment landscape, and also presents a forward-looking agenda for multi-dimensional research and policy in the decade ahead. It aims at providing a framework for future research that encourages cross-disciplinary collaboration as well as a structured dialogue between academia and policymakers.

## LONG-TERM TRENDS IN TRADE, INVESTMENT, AND GVCS

**Figure 1:**
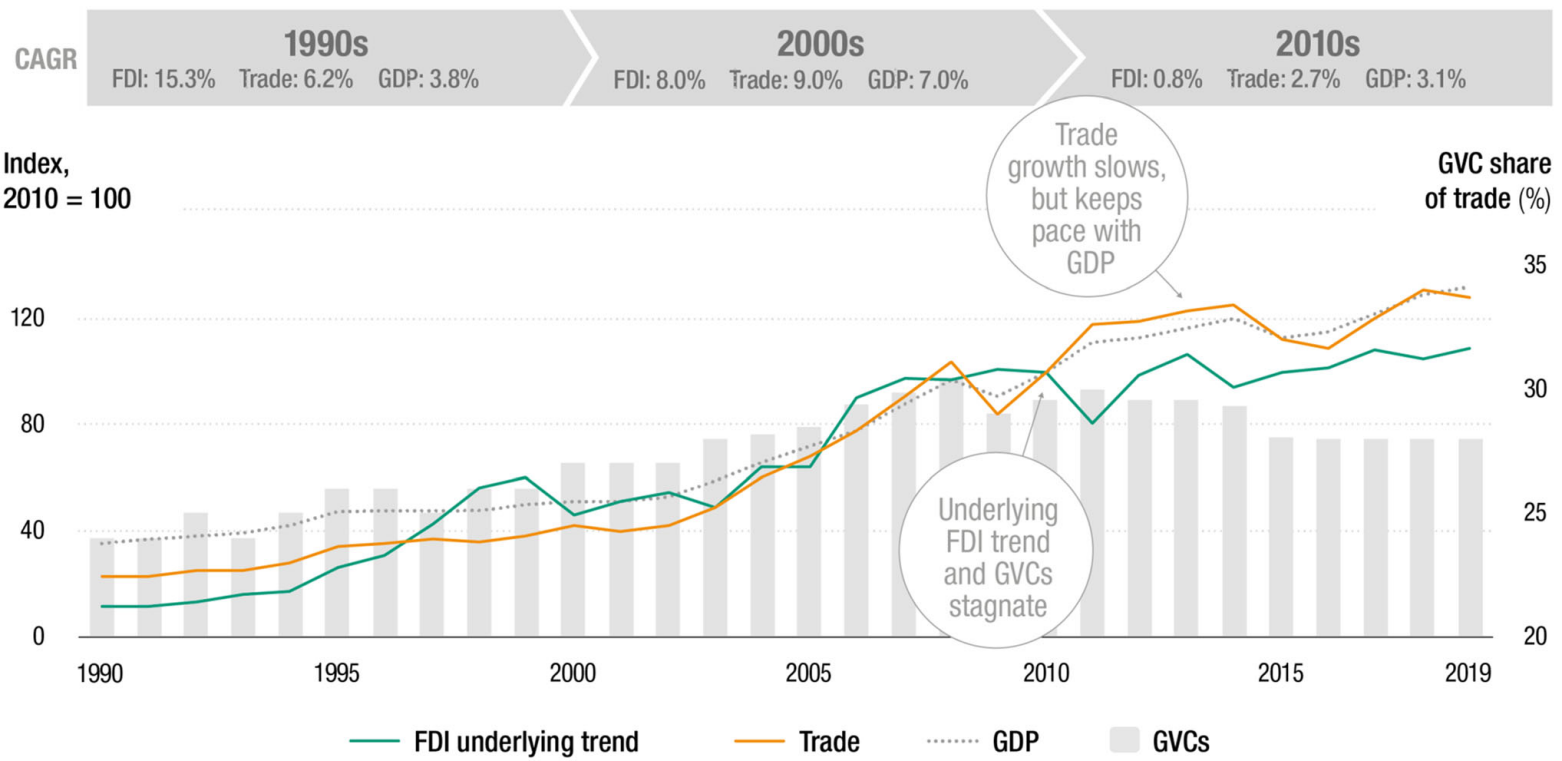
Global value chains: two decades of growth followed by one of stagnation (FDI, trade, and GDP indexed, 2010 = 100; GVCs percent). Source: UNCTAD (2020a)

During the past 30 years, international production has seen two decades of rapid growth followed by one of stagnation (UNCTAD, 2020a). Flows of cross-border investment in physical productive assets stopped growing in the 2010s, the growth of trade slowed down, and GVC trade declined as a share of total trade (see Figure 1).

In the early 1990s, the global presence of MNEs started to evolve from relatively simple cross-border structures predominantly motivated by the search for natural resources and international markets to more complex global value chains, built to exploit differences in labor costs and productivity.

In the 1990s and into the 2000s, this process accelerated. The two decades witnessed rapid growth in GVCs, a tenfold increase in the global stock of FDI and a fivefold increase in global trade. Roughly two-thirds of global trade was intra-firm trade between affiliates of the same MNE and trade within supply chains coordinated by MNEs.

A series of fundamental shifts in GVCs could be observed during this period. Patterns of FDI changed, with emerging markets becoming not only increasingly important recipients of FDI, but gradually also outward investors. The composition changed, with services playing a more important role, both through the internationalization of service industries and through the servicification of manufacturing activities, and the modalities through which MNEs expanded abroad changed, with mergers and acquisitions (M&As) playing a major role, and with corporate structures becoming highly complex.

In the 2010s, after the global financial crisis, the growth momentum of international production stalled. This was first reflected in trade. Worldwide exports of goods and services, which had grown at more than double the rate of GDP for decades, slowed down significantly relative to economic growth. The same development occurred in investment, even though the stagnation was obscured for some time by the expanding financial component of FDI. The data on investment flows net of conduits and offshore financial centers, clearly showed the lack of growth in global FDI.

It now seems that the 2010s were only the quiet before the storm. The crisis caused by the COVID-19 pandemic arrives on top of the pre-existing challenges to the system of international production (UNCTAD, 2020a). The decade to 2030 is likely to prove a period of transformation for global value chains, which will have significant implications for the global trade and investment landscape and MNEs’ modes of operation.

## FIVE DRIVING FORCES FOR GVC TRANSFORMATION TO 2030

Five major forces will drive the GVC transformation and reshape the global trade and investment landscape in the upcoming decade (Table 1).

1. *Economic governance realignment.* International economic policymaking and especially trade and investment policies are shifting away from multilateral cooperation towards regional and bilateral solutions. This is compounded by a general shift in national economic policymaking through new industrial policies, including protectionism in numerous countries (Zhan, 2019a). The aggravated systemic competition (trade, investment, technology, etc.) between economic powers may lead to a wide-spread global economic governance divide (Petricevic & Teece, 2019; Kobrin, 2020).
2. *The new industrial revolution.* The application of new technologies in international production of global MNEs will have far-reaching consequences for the configuration of GVCs. The key technology trends include robotics-enabled automation and AI-enhanced systems, supply chain digitalization (including platforms, cloud, Internet of Things (IoT), blockchain, and additive manufacturing and mass customization (UNCTAD, 2017; Brun, Gereffi & Zhan, 2019)). Each of these technologies will have distinct effects on the length, geographical distribution, and governance of GVCs. Each technology, depending on industry-specific deployment, will flatten, squeeze, or bend the “smile curve” of GVCs in its own way (UNCTAD, 2020a). All this will reshape the global trade and investment landscape and exert long-term social and economic impact in different parts of the world.
3. *Sustainability endeavor.* The big push for the green and blue agenda by markets and governments is changing products and processes along the value chains in the direction of sustainability (UNCTAD, 2014; UNCTAD 2018a; Zhan, 2019b; Zhan, 2020). According to UNCTAD’s latest estimates, the global sustainability-dedicated funds are now at the level of $1.2-1.3 trillion, including $260 billion of green bonds, $105 billion of social bonds, and over $900 billion sustainability-themed equity funds (UNCTAD, 2020a). The global effort to mobilize and channel investment to realize the Sustainable Development Goals (SDGs) will also change the future pattern of FDI, including the sources of financing, sectoral distribution, and geographical location (UNCTAD, 2015a; UNCTAD, 2020a). UNCTAD’s recent review has shown that six out of 10 SDGs groupings saw an increase in investment over the past 6 years. Investment in SDGs is expected to grow to trillions in the coming years.
4. *Corporate accountability.* International cooperation to fight corruption, illicit payments, tax evasion, and anti-competitive practices will have important implications for the modes of operation of MNEs. The environmental, social, and governance (ESG) standards and disclosure requirements will add to trade and investment policy pressures from both host and home states. Differences in approach between countries and regions on emission targets will impact the GVC governance choices. The author envisages the transformation of ESG standards in three dimensions in the coming years: A shift from icing-on-the-cake to integration into business models; from proliferation to harmonization of standards; and from voluntary to mandatory compliance.^1^. The transformation is gathering new momentum as governments and regulators are gearing up their efforts to integrate ESG into regulatory frameworks for FDI and capital markets, while MNEs worldwide are shifting their business value from shareholder-based to stakeholder-based (Zhan, 2020; UNSSE, 2019).
5. *Resilience-oriented restructuring*. The global crises, as well as growing geopolitical conflicts, will drive MNEs to make their global value chains more resistant to new types of shocks, and governments to reduce reliance on foreign sources for critical supplies. Admittedly, it will not be so easy for many MNEs to restructure their GVCs (Miroudot, 2020), and the reshoring and nearshoring have not been significant for some countries during the pandemic (Evenett, 2020). Nevertheless, the trend is emerging and likely to continue beyond the pandemic. MNEs will adopt new modes of business operations to enhance agility and flexibility, and to cope with increasing volatility, uncertainty, complexity, and ambiguity of the global business environment. All of this will exert a significant impact on the future pattern of global investment flows, including through diversification, redundancy and, to a certain extent, reshoring and nearshoring.

**Table 1:**
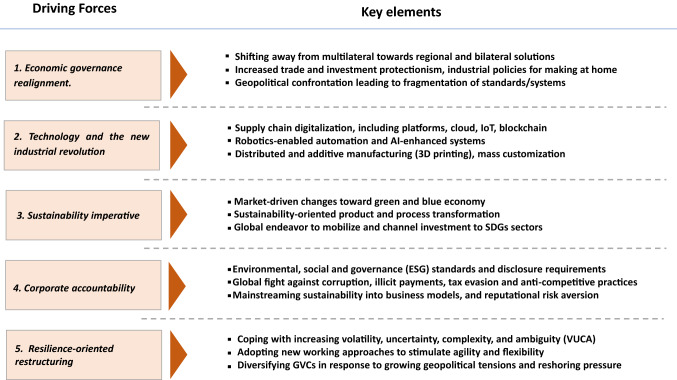
Five driving forces for GVC transformation and investment reconfiguration. Source: Author, partially based on UNCTAD (2020a)

The effects on global investment of the five driving forces are multifaceted. They are sometimes mutually reinforcing, and sometimes push in opposite directions. They play out differently across industries and geographies. Combined, these driving forces will impact three sets of locational determinants of the host countries: the policies and regulatory framework for international investment, the characteristics and dynamics of the host economy, and the investment facilitation and infrastructure.

The driving forces will also fundamentally alter the way firms across industries will design and operate their global value chains. They will influence MNEs’ strategic choices for international operations, i.e., modes of governance: arm’s length transactions (trade), FDI or non-equity modes. They will affect where MNEs choose to locate which type of activities, how they distribute value added, tangible and intangible assets over their networks, and how they transmit practices, including environmental, social, and governance practices, to actors along their value chains. Global patterns of trade and investment will change consequently, as will their potential impact on economic growth, employment creation, and sustainable development.

## FUTURE GVC TRANSFORMATION AND THE INVESTMENT LANDSCAPE: TEN DIRECTIONS

In light of the above five driving forces which will change the locational determinants and MNEs’ strategic choice for modes of operation, ten broad trends in the future GVC transformation and reconfiguration of global investment landscape can be projected in the coming decade (Table 2):

**Table 2:**
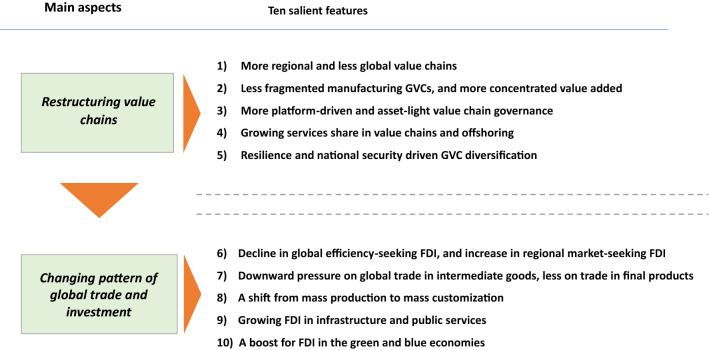
Ten salient features of the future GVC-investment landscape

### 1. More Regional and Less Global Value Chains

The predominant configuration of future value chains will be more regional than global. The regionalization of value chains can be the result of a pull-back from GVCs, with MNEs restructuring their global production networks into multiple regional and subregional production centers (relocation of value chain segments). The development of regional value chains can also be the result of the indigenous growth of international production in a region, with MNEs building new value chains at the regional and sub-regional levels (replication of value chains).

While regional value chains are not easy to establish and require regional coordination and conducive systemic conditions, the momentum for value chain regionalization is high and likely to grow further over the coming years, driven by the proliferation of regional integration initiatives (including regional free trade and investment agreements), pressure for post-pandemic resilience and regional self-reliance, aggravated geopolitical tensions, and the push for supply chain sustainability and responsibility. Rising protectionism may also further contribute to the regionalization of global value chains (Miroudot and Nordström, 2020).

### 2. Less Fragmented Manufacturing GVCs, and More Concentrated Value Added

The main direction of GVCs in modern manufacturing points towards simplification and localization, leading towards shorter and less fragmented value chains within an industry. The value added will tend to become more concentrated. The auto industry transforming from combustion engine to electrical vehicle manufacturing is a case in point. Production and value added are expected to become more concentrated because of the shift to electrical vehicles with far fewer components and shorter value chains. An average internal combustion engine has more than 2000 moving parts, while an electrical vehicle has only 20, with value added concentrated in few parts (UNCTAD, 2020a).

Distributed manufacturing will also lead to shorter value chains and a re-bundling of production stages with more geographically distributed activities, but more concentrated value added. Distributed manufacturing is generally associated with the application of 3D printing. Manufacturing models enabling replication range from networks centrally coordinated by MNEs to the bottom-up atomization of production whereby every firm independently produces final products. Digitalization of supply chains will enable a wider distribution of economic activities, but potentially with more concentrated value added in individual locations. Reshoring may also lead to reconfiguring some GVC segments, resulting in shorter, less-fragmented value chains and a higher geographical concentration of value added.

### 3. More Platform-Driven Governance and Asset-Light Value Chains

Digitally enhanced GVCs will strengthen the role of large digital MNEs providing the enabling digital infrastructure. These large digital MNEs tend to provide digital platforms concentrated in few economies and exhibit a distinctively “light” international footprint. Unlike traditional MNEs, digital MNEs possess fewer tangible foreign assets, indicating a reduced international physical footprint even though they generate a significant portion of their sales abroad (UNCTAD, 2017). In production-related investments by MNEs or their suppliers, other forms of investment may become more important for businesses with foreign asset-light business models, including knowledge-seeking foreign investments. For GVC governance, digital platform-based MNEs will complement, displace, or eventually lead to the adaptation of traditional MNEs as lead firms (Bolwijn, Casella & Zhan, 2018; Brun, Gereffi & Zhan, 2019).

**Figure 2:**
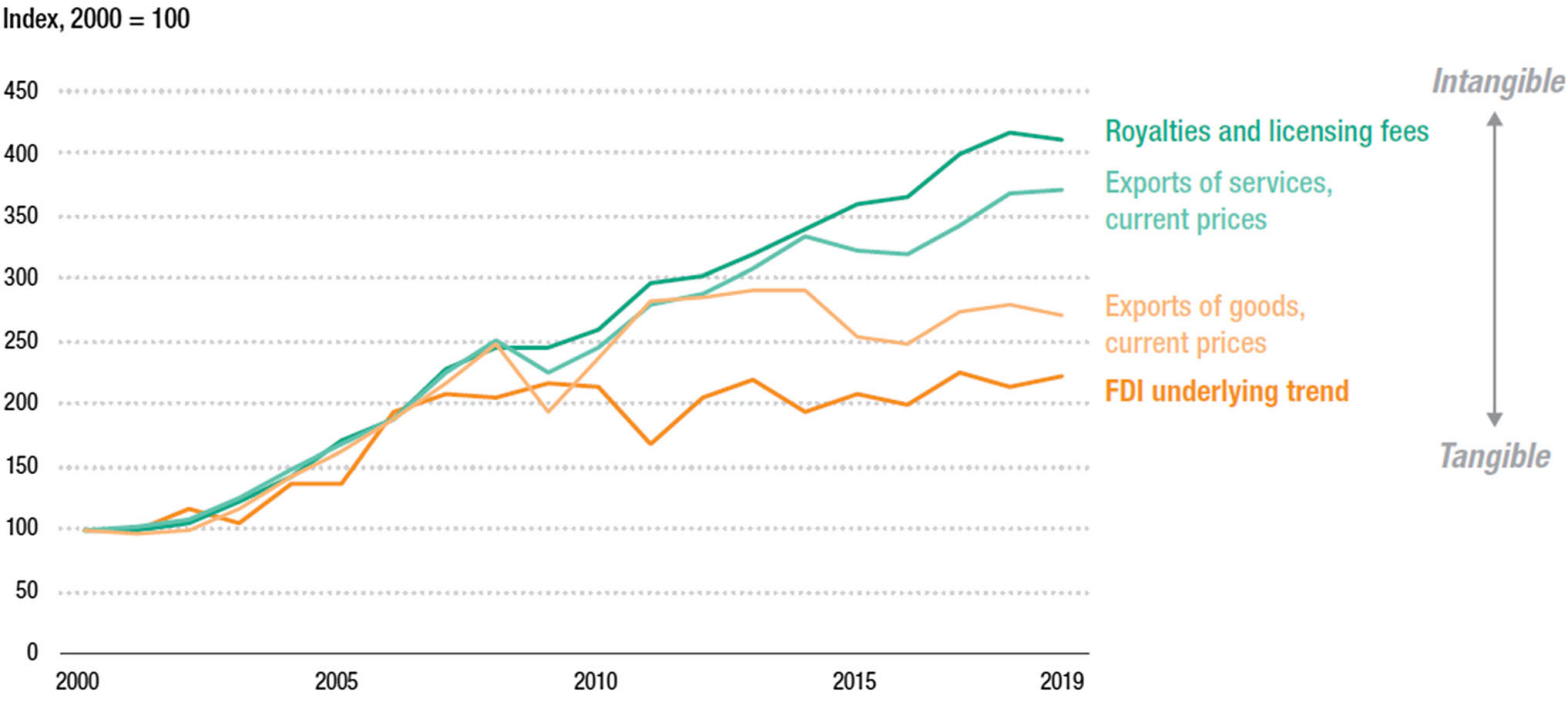
Indicators of international production by tangibility, 2000–2019. Source: UNCTAD (2020a)

The overseas operations of MNEs are becoming ever-more intangible and less dependent on investment in physical assets (Figure 2). Non-equity modes (NEMs) have become firmly established, between arm’s-length trade and FDI, as a governance mechanism in international production. NEMs allow MNEs to access overseas markets through contracts, rather than FDI, while still exercising a significant degree of control over operations. Technology-based MNEs also become increasingly important. These firms can reach markets worldwide through digital channels and without the need for a significant physical presence. The number of asset-light technology MNEs in UNCTAD’s 100 largest MNEs has increased, while manufacturing investment has declined (UNCTAD, 2017). At the same time, the value of greenfield cross-border investment projects in manufacturing industries was structurally lower (by 20 to 25%) than in the previous decade (UNCTAD, 2020a).

### 4. Growing Services Share in Value Chains and Offshoring

The broad application of enhanced digital technologies will seriously affect service industries, particularly higher value-added services – i.e., “white collar” services, ranging from professional and business services to finance, engineering, and marketing activities. The enhanced digital technologies could make service industries the new frontier of offshoring, driven by labor cost arbitrage. High and medium value-added services, traditionally highly centralized, will be increasingly delivered offshore through teleworking. Teleworking opportunities are being enhanced by advanced digital communication tools, including teleconferencing, augmented reality, virtual reality, and 5G. Cloud storage and computing make it possible to perform complex tasks remotely, while improvements in translation software will facilitate communication (Baldwin, 2019).

Expansion of services GVCs will also be boosted by the unbundling of manufacturing-related activities into services, a process known as servicification of manufacturing (UNCTAD, 2017; Brun, Gereffi & Zhan, 2019). The impact of digitalization on servicification is twofold. On the one hand smart manufacturing driven by the Internet of Things and Big Data increase the service content of the final product, for example in terms of digital and ICT services (embodied services). On the other, new services are added to the final product, generally with a major digital component (embedded services, such as software upgrades for cars and washing machines). These transformations will lead to a hybrid, highly fragmented environment where manufacturing activities are increasingly integrated with digital services (UNCTAD, 2020a; Bolwijn, Casella & Zhan, 2018).

### 5. Resilience and National Security-Driven GVC Diversification

One trajectory to build resilience in GVCs is diversification and redundancy. As the perceived over-concentration of production and supply chain dependence are of primary concern by many governments, firms and states may find that diversifying internationally can be more effective than reshoring (and *de facto* reconcentrating domestically). This means giving up some economies of scale by involving more locations and suppliers in the value chain. Digitalization of the supply chain is pivotal to the process of diversification, as much as automation is the technological trigger of reshoring. Firms in many GVCs will have the opportunity to maintain and potentially extend their complex network of international operations by leveraging digital technologies to improve coordination and control. The mix of control over the supply chain, enhanced marketing and flexible processes enabled by a smart integration of digital technologies allows firms to prevent, or at least manage, the kind of supply, demand, and operational shocks that were so dramatically showcased by the COVID-19 pandemic.

### 6. Decline in Global Efficiency-Seeking FDI, and Increase in Regional Market-Seeking FDI

The impact of digitalization and automation on labor cost differentials will exert downward pressure on global efficiency-seeking FDI. Distributed manufacturing enabled by IoT and 3D printing potentially favors an increase in market-seeking FDI. Like robotics, 3D printing reduces the labor component in production and the competitive advantage generated by labor cost differentials. The transition from efficiency-seeking, vertically specialized, value chains to distributed market-seeking manufacturing is also favored by relatively limited capital cost differentials across countries. Overall, the weight of factor cost differentials in internationalization decisions becomes smaller (UNCTAD, 2020a).

### 7. Downward Pressure on Trade in Intermediate Goods, Less on Trade in Final Products

As GVCs in manufacturing become more local, shorter, and less fragmented, physical concentration of the production process will decrease cross-border trade in intermediate inputs and components. While reduction in intermediate trade is already happening and it is likely to accelerate in the future, the prospects for trade in final goods are less obvious.

Transformation of some industries will also reduce the production and trade in intermediate goods. As explained above, the transformation of the auto industry from combustion engine to electrical vehicle manufacturing is a good example. Due to platform sharing and the shift to electrical vehicles with far fewer components, the value chain will become shorter and the value added more concentrated. Consequently, electric vehicle supply chains will involve far fewer suppliers and much less trade in intermediate goods.

### 8. A Shift from Mass Production to Mass Customization

Advanced manufacturing points to a reconfiguration of international production characterized by small-scale, localized production, close to the point of consumption. It is also supported by the application of new production technology – such as 3D printing (UNCTAD, 2017). 3D printing changes the traditional patterns of international production by the effects of re-bundling through technological inseparability.

Distributed manufacturing enables the shift from mass production and economies of scale to mass customization. Value added stems from the design/programming phase – delivering the specifications for replicable 3D printing – and the customer-related activities, addressing the clients’ needs. The manufacturing step tends to be a highly commodified, low value-added but capital-intensive activity replicated in multiple countries. The focus and source of value switches then from economies of scale to economies of scope (UNCTAD, 2020a). The pharmaceutical industry is an example of the application of distributed manufacturing (“hub and spoke”). Beyond pharmaceuticals, distributed manufacturing may have applications in customized segments of mass industries such as apparel or food that are characterized by limited production complexity.

The servitization of manufacturing to seek economies of scope can also lead to the transformation to customization. According to Baines et al. (2017), servitization is defined as a process of building revenue streams for manufacturers from services. In addition to basic and intermediate services traditionally offered (such as spare parts, product repairs, maintenance, helpdesks, training, condition monitoring), manufacturers now also provide advanced customized services, such as customer support agreements and outcome contracts. Examples of companies delivering such advanced services include Rolls-Royce, Caterpillar, Alstom, MAN, and Xerox (Baines et al., 2017).

Services have also been described as value-creating activities, as their role is to create value all along the GVC. Services lead to higher value creation and are part of a shift towards more productive and more customer-centric production models where value can be seen as co-created with consumers (Demirkan et al., 2011).

Supported by increasing Big Data and IoT, servitization of manufacturing will exert a far-reaching impact on future trade and investment by MNEs.

### 9. Growing FDI in Infrastructure and Public Services

Large amounts of capital are now looking for investment opportunities for value-creating projects in infrastructure, agriculture, and public services. Some services that have always been predominantly domestic are internationalizing, such as health care, education, and digital infrastructure, just as traditional international production industries are retreating or restructuring. That creates new opportunities for promoting investment in new areas (UNCTAD 2020a; UNCTAD 2018a). In the short to medium term, the development of infrastructure may support domestic recovery by boosting the local economy and employment. In the medium to long term, investment in infrastructure enables building more resilient local and regional ecosystems, physically and digitally integrated, as well as supporting sustainable growth.

### 10. A Boost for FDI in Green and Blue Economies

Driven by the global sustainability imperative, promoting the green economy and the blue economy (e.g., renewable energy, land and water management, sustainable maritime sector, climate change mitigation and adaptation) presents great potential for international investment. MNEs will increasingly align their investment decisions, production processes, and products and services with the sustainable development agenda. Harmonization of ESG standards and strengthening of corporate accountability will exert further pressure on MNEs and their global supply networks to deliver sustainable impact through investment. As the green and blue economies gain priority on the political agenda, they will benefit from massive policy support, including through the mainstreaming of the SDGs into investment policy-making and the reorientation of investment promotion and facilitation strategies towards the SDGs.

## A FRESH RESEARCH AND POLICY AGENDA FOR THE DECADE AHEAD

As the transformation is unfolding, it is time to chart the way forward in research and policy analysis to address tomorrow’s investment-development challenges and opportunities.

In the previous section, ten broad trends in GVC transformation and the global trade and investment landscape have been highlighted (Table 2). The ten broad trends can serve as a basis for a forward-looking research agenda for international business (IB) scholars and scholars in adjacent disciplines, such as international finance and economics, international investment law, and development studies. Each of these trends merits more in-depth research, and perhaps necessitates new theory. At the minimum, these trends require close monitoring throughout the process of the transformation. Empirical studies can help validate, update, enrich and fine-tune established theoretical frameworks for international production systems and GVCs.

Furthermore, the five driving forces and ten trends can also present a long list of topical issues for evidence-based policy analysis on GVC-development strategies in the coming decade (Zhan, 2020). Three main dimensions of the policy analysis agenda are highlighted below: first, the investment policy core – priorities for policy action; second, policy implications of the five driving forces – addressing the key emerging trade and investment issues; and third, international investment and global crises – a challenge for building back better.

### 1. The Investment Policy Core – Priorities on the Agenda

There is a need to continue the great endeavor embarked on at the time of the investment-development paradigm shift (Zhan, 2010). In the past decade, considerable policy analysis has focused on modernizing national laws and regulations and formulating a new international investment treaty regime (UNCTAD, 2015b; Zhan, 2020) whose overarching objective is to align investment policies and governance with sustainable development at the national and international levels. Investment policies typically address five sets of core regulatory issues, i.e., liberalization, protection, promotion, facilitation and dispute settlement (UNCTAD, 2015a; UNCTAD, 2017). Four priorities are highlighted by the author below.

### 1) Updating Industrial and Investment Policies in the Era of GVC transformation

Industrial policies have been proliferating. UNCTAD’s global survey of industrial policies shows that today over 100 economies, both developed and developing, have adopted formal industrial development strategies. Together, they account for more than 90% of global GDP. The last few years have seen an acceleration in the formulation of new strategies (UNCTAD, 2018a).

Modern industrial policies are increasingly diverse and complex, addressing new themes and including myriad objectives beyond conventional industrial development and structural transformation. Investment policies (in particular in developing countries) have been a key instrument of industrial policies. Different industrial policy models come with a different investment policy mix.

Modern industrial policies need to follow several design criteria. These include openness, sustainability and readiness for the technological revolution. Investment policy choices should be guided by these design criteria and by the need for policy coherence, flexibility, and effectiveness. Industrial policies need to use different investment policy tools and focus on different sectors, economic activities, and mechanisms to maximize the contribution of investment to the development of industrial capabilities. The investment policy toolkit needs to be developed in line with industrial policy models and stages of development.

**Figure 3:**
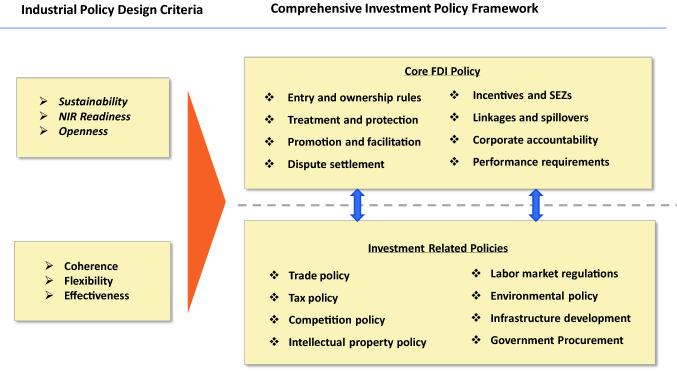
Industrial policy design and investment policy framework.

Governments need to ensure that their investment policy instruments are up-to-date, including by reorienting investment incentives, modernizing SEZs, retooling investment promotion and facilitation, and crafting smart foreign investment screening and monitoring mechanisms. The transformation of GVCs requires a strategic review of investment policies for industrial development. For modern industrial policies to contribute to sustainable development, policymakers need to enhance their coherence with national and international investment policies and other related policy areas, including trade, tax, and competition (Owens and Zhan, 2018), as well as social and environmental policies (Figure 3). They need to take a “whole of government” approach to create synergy. They also need to strike a balance between the roles of the market and the state and to avoid overregulation. Finally, they need to adopt a collaborative approach that is open to international productive capacity cooperation and avoid beggar-thy-neighbor outcomes (Zhan, 2011).

As industrial policy further proliferates and becomes the mainstream of development strategy, a key challenge is emerging today: new industrial policies need to make more effective use of investment policy instruments, and investment policies need to modernize in line with new industrial development strategies. Together, they need to be updated to address the key emerging challenges identified in the previous section.

### 2) Boosting Investment in SDGs

A second top priority today is to mainstream SDGs into their national policy framework and investment promotion strategies. More than 150 countries have adopted national strategies on sustainable development or revised existing development plans to reflect the SDGs. Nevertheless, an analysis by UNCTAD, based on 128 voluntary national reviews, reveals that although many of these strategies highlight the need for additional financial resources, very few contain a concrete roadmap for the promotion of investment in the SDGs (UNCTAD, 2020a).

Existing investment promotion instruments applicable to the SDGs are limited in number and follow a piecemeal approach. UNCTAD’s global review of national investment policy regimes shows that less than half of UN Member States maintain specific promotion tools for investment in the SDGs for these countries, and on average, each targets no more than three SDG-related sectors or activities in its regulatory framework. Many other countries do not have such policies at all. Countries promote inward investment in the SDGs primarily through incentive schemes. Several key SDG sectors, such as health, water and sanitation, education, and climate change adaptation, are rarely covered by specific incentives.

A more systematic approach is needed for mainstreaming SDGs into national investment policy frameworks, and factoring investment promotion into national SDG strategies (Zhan and Paulino, 2021).

### 3) Reforming the International Investment Treaty Regime

**Figure 4:**
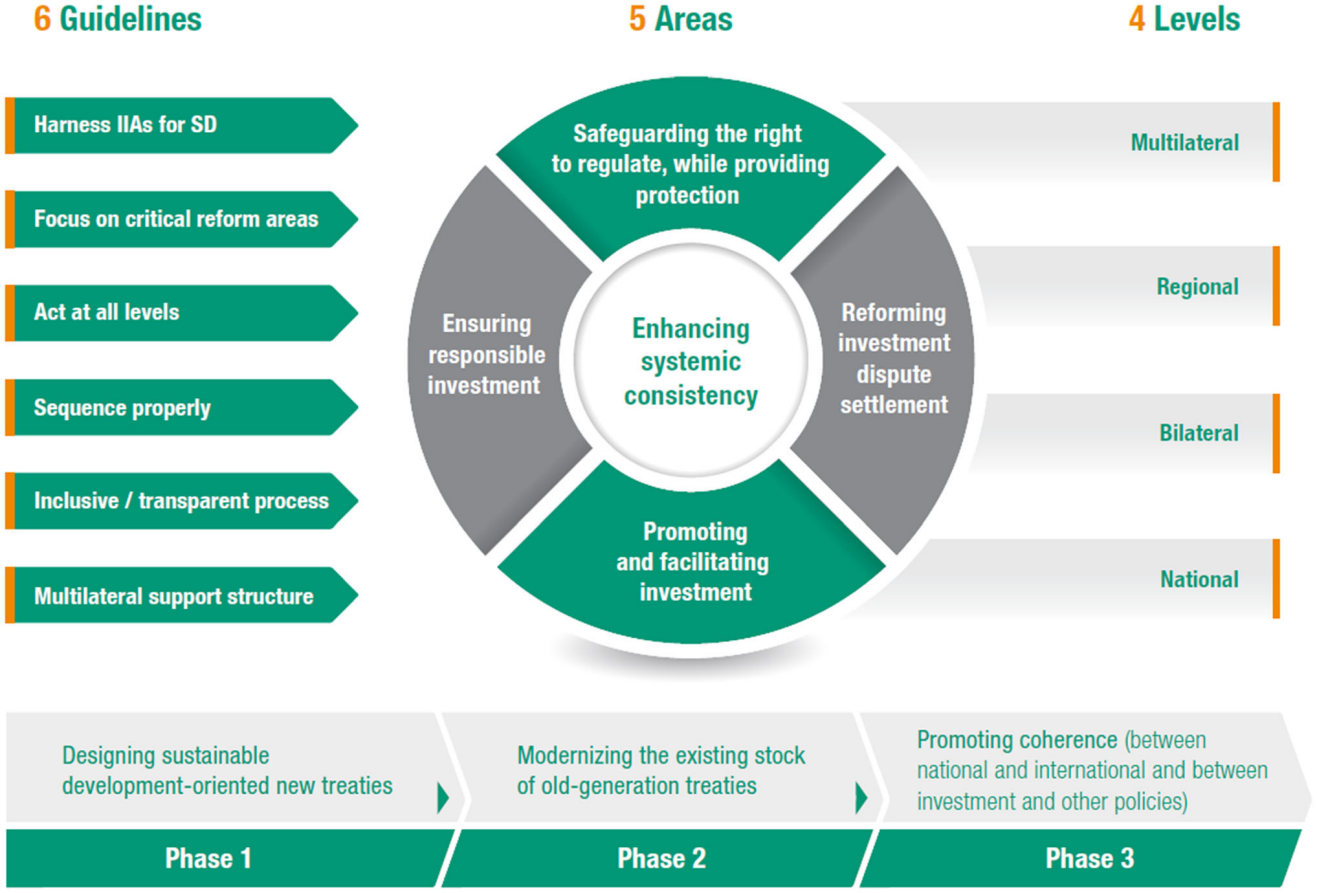
Roadmap for reforming international investment treaty regime. Source: UNCTAD (2017b)

Reforming the international investment agreement (IIA) regime is also a top priority. UNCTAD advocated the reform of the IIA regime, and drew the road map for IIA reform, covering in a single package all key aspects of the reform (UNCTAD, 2017b; Zhan, Weber & Karl, 2011). It strategizes the reform by three phases, at four levels, in five key areas, and under six guiding principles (Figure 4).

The five core areas for reform include enhancing systemic consistency, safeguarding the right to regulate while providing investors protection, ensuring responsible investment, promoting and facilitating investment, and reforming investment dispute settlement mechanism. So far, significant progress has been achieved, particularly with respect to the new generation of IIAs. Almost all the newly concluded IIAs have factored in the core elements contained in UNCTAD’s reform package. Nevertheless, reform of the existing stock of the 2500 old-generation bilateral treaties still in force today has not yet taken off on a large scale, while the number of known treaty-based ISDS cases continues to grow. Most of these cases are triggered by the old-generation treaties, thereby increasing the risk against State measures in pursuit of legitimate public policy objectives. In line with the Reform Roadmap, UNCTAD has just introduced the “IIA Reform Accelerator”, focusing on expediting the modernization of the existing stock of IIAs (UNCTAD, 2020b).

The importance of reforming the stock of old-generation treaties is further highlighted by the COVID-19 pandemic and the transformation of GVCs. IIAs can place constraints on the policy responses taken by governments to address the pandemic and the economic fallout in the short term and transformation of GVCs in the longer term, because these government measures also affect the operations of foreign investors.

Modernizing the existing 2500 old-generation IIAs and mainstreaming sustainable development into the future generation of IIAs remain a daunting task ahead of the international investment community.

### 4) A Soft Approach to Multilateralism for Investment

At the global level, investment policy making has been handicapped by the absence of a multilateral investment system. The global economic governance is organized around several institutional pillars, including the IMF that oversees the multilateral monetary system, and the WTO that coordinates the multilateral trade system, but there is no equivalent for the international governance of investment. The current *de facto* regime is governed by thousands of bilateral and regional agreements, and other ad hoc treaties and rules. In the absence of a multilateral intergovernmental system for formulating global rules for cross-border investment, a multi-stakeholder consensus-building approach can be effective and impactful (Zhan 2019a; Zhan, 2020).

The idea is to supersede the conventional approach to global investment policymaking – intergovernmental negotiations – by providing a platform for inclusive and comprehensive dialogue among the whole community of investment-development stakeholders that would gradually lead to consensus on investment policies and norms. We at UNCTAD envisaged a virtuous cycle of policymaking, from proposals and policy options by the UNCTAD Investment Division and our global investment network, through multi-stakeholder deliberations and consensus building at the World Investment Forum, to policy implementation in the field supported by technical assistance, to feeding back lessons learnt into further policy research and formulation.

This has proved a viable and pragmatic approach to shaping a new generation of international investment policies – a “soft approach to global investment policymaking” as I coined it, as opposed to the hard negotiations-based approach. Ultimately, multilateral rules and principles are meant to be translated into national laws, regulations, and policy practices of individual countries. The “soft approach” can achieve the same objective without hard bargaining, and our experience suggests this is the case, as the examples below attest. After all, the investment relationship is a long-term partnership, rather than a give-and-take deal. Furthermore, our soft approach represents the “best endeavor”, while a negotiation-based approach may end with the “lowest common denominator” in standards (Zhan, 2019a).

The “soft approach” has proven successful and can be more effective than a traditional approach to negotiations. Concrete examples abound (UNCTAD, 2019; Zhan, 2020; Sauvant, 2020). For example, between 2012 and 2016, 148 countries reviewed their national and/or international investment policies, with 133 of them using UNCTAD’s policy guidance in the Investment Policy Framework for Sustainable Development (UNCTAD, 2015b) for that purpose.^2^ The G20 Guiding Principles for Global Investment Policymaking drew on UNCTAD’s Investment Policy Framework for Sustainable Development (Zhan, 2016). The G20 Guiding Principles are the first multilateral consensus on investment matters reached among a group of developed, developing and transition economies, accounting for over two-thirds of global outward FDI. Other examples include the UN Sustainable Stock Exchange Initiative (UNSSE, 2019), Global Compact Initiative and OECD policy instruments.

As multilateralism is further eroded and a multilateral investment system continues to be absent, more ways and means need to be identified to strengthen multilateral cooperation in investment for sustainable development.

### 2. Big questions: Policy implications of the five driving forces

The five major driving forces (Table 1) explained earlier will also reshape the global trade and investment policy space, and business ecosystem in general, in terms of norms, strategies, rules and regulations, as well as administrative practices. New investment-development policy implications call for a new policy analysis agenda. Towards this end, I sketch out a general framework with five sets of key questions below (the following issues are illustrative, not exhaustive).

#### 1) Global economic governance realignment and investment policies

- What are the implications of the GVCs’ transformation for the international trading system and investment treaty regime, and how to address the emerging challenges arising from the realignment of global economic governance?
- How can we foster policy coherence for cross-border investment in light of the growing fragmentation in international trade and investment policymaking? What types of multilateral platforms and soft instruments can be employed?
- How can we effectively monitor global investment trends and policy development, with particular attention on growing protectionism and concerns of national security?
- What are the implications of systemic competition between economic powers for FDI flows to developing economies?
- How can we safeguard a stable, predictable, and transparent regulatory framework for cross-border investment in the era of VUCA (volatility, uncertainty, complexity, and ambiguity)?

#### 2) The new industrial revolution and investment-for-development strategy

- How can we address the key emerging challenges and opportunities for countries at a time of transformation and re-configuration of GVCs by MNEs?
- What type of new strategies should host countries put in place to promote and facilitate investment in new sectors and new business models for growth and prosperity?
- How can we close the technology divide between advanced and the least-developed countries?
- How can we create synergies among industrial policy, investment policy, and technology policy?

#### 3) The sustainability endeavor and international production

- How do we mobilize and channel investment into the 17 SDG sectors? What should be done to improve the capacity of developing host countries in preparing bankable project pipelines?
- How can we promote and facilitate international investment to green and clean traditional value chains?
- What are the implications of climate change adaption and mitigation measures for the operations of global value chains and investment strategy of developing countries?
- How can we maximize FDI and MNE contributions to inclusive growth, addressing the challenges of poverty and inequality, including gender and race?

#### 4) Corporate accountability and business implications

- How do sustainability-related rules, regulations, and standards impact GVCs and patterns of FDI?
- How can we harmonize the myriad of ESG standards and foster effective sustainability reporting and mobilization of sustainable investment?
- How can business contribute to international cooperation in fighting corruption, illicit payments, and tax evasion?
- How can we mainstream ESG into business models and the operation of GVCs?

#### 5) Business restructuring for resilience and policy implications

- How can countries cope effectively with the impact of the pending reconfiguration of international production, including divestment, diversion, and diversification?
- How can countries shift investment policy direction from a GVC towards an RVC (regional value chain)-based approach?
- How can developing countries, used to attracting export-oriented investment, more effectively promote international investment in infrastructure, services, and domestic manufacturing capacity?

### 3. International Investment and Global Crisis

Additional to the above five sets of big questions and change processes is the issue of international investment and global crises – a challenge for building back better. The world has witnessed at least one major global crisis per decade and a myriad of smaller ones in between. Many more are coming. It is imperative to learn the lessons from the past and current major crises for future business strategies and policy responses (Zhan and Ozawa, 2001).

Global crises, e.g., food, fuel, financial, pandemic, or geopolitical, usually create triple shocks, i.e., economic, social, and policy shocks, with significant implications for global investment. In the current context, fighting the worst economic downturn in living memory, policymakers around the world have responded forcefully. The fiscal support of over $12 trillion has eclipsed previous records. Monetary easing, liquidity injections, and asset purchases have helped prevent financial catastrophe. Nevertheless, they are by no means the magic key for a sustainable global recovery. In fact, the world is in a global liquidity trap, which governments and central banks will sooner or later have to find a way to climb out of. Debt relief is helpful for developing countries to reduce the financial burdens and unlock some domestic financial potential, but it does not resolve the critical issue of a grand sustainable recovery. It is time to focus on the real economy. Restarting the engine of global production and strengthening productive capacity are key at a time when the world is moving from crisis response to sustainable recovery and inclusive growth. All this needs investment in real sectors. Among the key questions are:

- What types of strategies and policy measures are required to re-start the engine of international production for economic recovery and reconstruction?
- For lessons learnt, how do crises impact global investment flows; and how do MNEs react to global crises and to the massive policy interventions?
- How can we promote investment to build back better, i.e., towards a resilient and sustainable economy?

It is time for a global synchronized push for investment in sustainable recovery. Without productive investment, there will be no building back better.

The critical policy agenda above presents the research topics commonly tackled within international investment law, international economics and finance, and development studies. Nevertheless, they are also directly relevant for international business (IB) scholars. These issues affect business and are affected by business. Therefore, tackling these issues will enable IB research at the firm and industry level not only to provide sound advice to MNEs for operating in a rapidly evolving global business ecosystem, but also to contribute insights that are relevant and novel for policymaking in the global context (Zhan, 2010; Zhan, 2020).

Peter Buckley et al. (2017) called for a redirection of IB research toward big questions and “grand challenges” in global business and phenomena-driven perspectives to address those questions. Indeed, IB can and should play a more constructive and vital role by tackling expansive topics at the business–society interface (Buckley, 2020; Eden, 2020; Zhan and Mirza, 2012).

## CONCLUSION

This article has addressed the transformation of global value chains and a new investment landscape leading to 2030, highlighting five driving forces and ten broad trends. It provides a framework for a forward-looking multi-disciplinary and multi-dimensional research and policy analysis agenda for the 2020s and beyond. It is meant to help in shaping the future research orientation, facilitating cross-disciplinary collaboration, as well as stimulating dialogues between academia and policymakers in line with the objectives of the *Journal of International Business Policy* (Lundan, 2018; Van Assche, 2018), and the journal *Transnational Corporations* (UNCTAD, 2018b).

Global value chains will undergo a transformation in the decade ahead. The evolution will be driven by five major forces: Economic governance realignment, the new technology revolution, the sustainability endeavor, the corporate accountability push, and resilience-oriented restructuring. These forces impact on the locational determinants and MNEs’ strategic choices for international operations, and subsequently reshape global investment landscape in at least ten broad directions. All this will present daunting challenges and ample opportunities for firms and states alike, leading to an investment-development paradigm shift.

For the policy agenda, the big question as to where global trade and investment policy is heading in the 2020s is still open, but a new dichotomy in policy direction is emerging: We are entering a decade of de-globalization and the mainstreaming of sustainability. The two policy trends are somewhat incompatible. The former leads to further fragmentation of the global market and erosion of multilateralism, while the latter depends on the establishment of global standards, global governance, and global partnerships (Zhan, 2019b).

All this implies a change in investment policy thinking from the international production networks that have been the core focus of IB research towards the need to attract international investment aligning with the Sustainable Development Goals. This will further necessitate scholars to engage in inter-disciplinary research, combining perspectives from international business, international finance and economics, international investment law and development studies to address broader critical challenges for investment and sustainable development. It is also important for scholars to monitor closely the rapid and massive policy developments and analyze their implications for MNEs’ international investment and operations. The two academic awards, i.e., UNCTAD-AIB Award^3^ and UNCTAD-SIEL Award^4^ for the best young academic research work on investment and development, which the author initiated in 2019 in collaboration with AIB and the Society of International Economic Law, are aimed at inspiring the new generation of scholars to direct their research efforts towards investment and sustainable development in the 2020s and beyond.

## Notes

1. Author’s opening statement, in his capacity as chairperson of the UN SSE Governing Board, at the Conference on the 10th Anniversary of the UN Sustainable Stock Exchanges Initiative, 26 September 2019, at the New York Stock Exchange. https://sseinitiative.org/wp-content/uploads/2019/12/SSE-10-year-impact-report.pdf.
2. See UNCTAD (2019) (and earlier editions) for documentation of the results and impact of UNCTAD’s soft normative work and advisory services for over 130 countries and regional groupings.
3. For UNCTAD-AIB Award, see https://www.aib.world/about/awards/unctad-aib-award-for-research-on-investment-and-development/.
4. For UNCTAD-SIEL Award, see https://www.sielnet.org/prizes/siel-unctad/.
